# Empathy and teachers’ fairness behavior: The mediating role of moral obligation and moderating role of social value orientation

**DOI:** 10.1371/journal.pone.0268681

**Published:** 2022-06-09

**Authors:** Youjuan Hong, Jingxue Cai, Ruiming Lan, Kaixuan Wang, Rong Lian, Lijun Chen

**Affiliations:** 1 School of Nursing, Fujian Medical University, Fuzhou, China; 2 School of Education, Fujian Polytechnic Normal University, Fuqing, China; 3 School of Psychology, Fujian Normal University, Fuzhou, China; 4 Department of Student Affairs, Fujian University of Traditional Chinese Medicine, Fuzhou, China; 5 School of Humanities and Social Sciences, Fuzhou University, Fuzhou, China; Polytechnic Institute of Coimbra: Instituto Politecnico de Coimbra, PORTUGAL

## Abstract

This study examined the mediating effect of moral obligation and moderating effect of social value orientation on the relationship between empathy and fairness behavior in Chinese teachers. Seven hundred and twenty-six Chinese teachers completed self-reported questionnaires regarding empathy, moral obligation, social value orientation, and fairness behavior. The results revealed that moral obligation mediated the link between empathy and teachers’ fairness behavior. Teachers’ social value orientation moderated the associations between empathy and moral obligation and moral obligation and fairness behavior. The associations between empathy and moral obligation and moral obligation and fairness behavior were more robust for those with high SVO scores (i.e., prosocial). This study identified the critical factors associated with teachers’ fairness behavior, supplying empirical support for existing theories and providing practical implications for interventions designed to improve Chinese teachers’ classroom environment.

## Introduction

Teachers’ fairness behavior refers to their treating students equally, without being affected by factors such as level of academic achievement or family background. Fairness is a basic psychological need [1]. However, students from many countries have reported experiencing unfair treatment at school [2,3]. For instance, certain groups of pupils (e.g., high performers) are often treated better and with more respect than others [4,5]. Teachers’ fairness behavior is an important factor in students’ physical and mental health [6]. Evidence accumulated over the past two decades has shown that a lack of fairness in the classroom can predict students’ motivation to learn, the teacher-student relationship [7], and maladaptive behaviors such as bullying, Internet addiction, and leaving school before graduation [8].

However, prior studies of teachers’ fairness have been conducted from the perspective of the students, and not the actual actors. In the field of education, it is often teachers who initiate acts perceived by students as fair or unfair. At present, little attention has been paid to factors affecting teachers’ fairness behavior. Thus, a clear understanding of the motives underlying such actions is necessary [9,10]. When it comes to elements that shape prosocial behavior in interpersonal relations, few psychological constructs can compare with empathy [11]. Whiteside and Barclay found that as an emotional factor, empathy is an important antecedent of fairness [12]. Whether empathy plays a role in fairness (and if so, by what mechanisms) has yet to be studied from the perspective of educators, especially when considering the individual variables of moral obligation and social value orientation (SVO). Therefore, the goals of the present study were to test the hypothesis that empathy plays a role in teachers’ fairness behavior and explore the mediating role of moral obligation and moderating role of SVO.

## Literature

### Empathy and teachers’ fairness behavior

Empathy has been defined as the ability to identify what someone else is thinking or feeling and respond to those thoughts and feelings with an appropriate emotion [13]. Empathy, a quality considered desirable in a teacher, is a key educational resource for achieving social justice [14,15]. The empathy-altruism hypothesis argues that a person’s empathy can positively predict their prosocial behavior, including their ability to be fair [16]. For instance, nursing students’ empathy was significantly and positively related to their tendency to be fair [17]. Patient and Skarlicki found that managers with low levels of empathy behaved less fairly towards their subordinates than did those who were more empathetic [18].

Empathy plays an important role in fairness because it can increase an individual’s perception of its importance. Specifically, individuals with greater levels of empathy are better at understanding the negative outcomes that unfairness may have for others; they realize that they should treat others in a fair manner in order to minimize the harm that not doing so might cause [19]. In an educational context, teachers’ empathy is an important factor influencing teacher-student interactions [20]. Empathy prompts concern for a distressed person’s welfare, and in turn motivates a desire to find ways to improve their situation. Teachers with high levels of empathy have a more positive attitude regarding special students (e.g., those with low academic achievement); they are more aware of their needs and willing to build close relationships with them [21]. For instance, highly empathetic teachers have a better attitude regarding undocumented immigrant students [22]. Consequently, such teachers seek to improve students’ lives through fairness [23]. Research has found that teachers’ empathy affects their ability to treat their students fairly. For example, pre-service teachers who showed empathy were found to be better able to understand and practice social justice in their teaching [24]. When researchers found ways to increase teachers’ level of empathy, White teachers’ implicit bias towards Black students decreased and differential treatment was reduced when disciplinary problems arose [25]. Thus, we propose the following hypothesis.

**Hypothesis 1.** Empathy positively predicts teachers’ fairness behavior.

### Moral obligation as a mediator

The deontic theory of justice has proven valuable for examining the motivation to behave fairly [26]. This theory states that individuals are motivated to seek fairness based on a sense of responsibility, duty, and moral virtue, which together emphasize that everyone should be treated justly [27]. The function of attitude (e.g., fairness obligation) in recipients of fairness may also be found in actors. Individuals with a greater sense of moral obligation feel more responsible for maintaining fairness norms, show more anger in response to unfairness, and thus are more likely to intervene when witnessing unfair events [28,29].

Researchers have found that empathy is an important factor influencing individual moral obligation [30]. Empathy may increase teachers’ sense of moral obligation, and thus their tendency to treat students fairly. The empathy-altruism hypothesis suggests that when highly empathetic individuals are aware that others are being treated unfairly, they automatically perceive suffering and experience similar emotions, often prompting them to react as if the injustice is happening to them. Research has also found that highly empathetic individuals have a greater ability to perceive unfairness, show more anger in response, and are more motivated to oppose it [31]. Therefore, teachers’ empathy helps them to be cognitively aware of the harm caused by unfairness, leading them to emotionally experience the pain of unfair treatment and making it easier to feel morally obligated to treat their students fairly.

Moreover, the deontic theory of justice suggests that increasing a person’s sense of obligation to be fair can play an important role in promoting fairness behavior; that is, a sense of moral obligation can inspire individuals to treat others fairly. Such a moral obligation leads to unease when perceiving injustice to others, inspiring acts of resistance [32]. Research has also shown that individuals motivated to be fair are more likely to initiate and persist in fairness efforts [33]. Because a sense of moral obligation inspires teachers to feel responsible for being fair, those with a greater sense of such obligation are less likely to treat students differently. Thus, highly empathetic teachers are less likely to show preferential behavior. Consequently, we propose the following hypotheses.

**Hypothesis 2.** Moral obligation is positively related to empathy and fairness behavior.
**Hypothesis 3.** Moral obligation mediates the effect of empathy on fairness behavior.

### Social value orientation as a moderator

The positive impact of empathy on fairness obligation is also influenced by interpersonal traits such as SVO. SVO refers to preferences affecting the allocation of resources between oneself and others. Two main types are commonly distinguished [17]. Persons with prosocial tendencies are willing to establish equal allocation and/or maximize mutual benefit [34], while individuals with pro-self tendencies focus on their personal benefit, trying to maximize their own resources [35]. As a personality trait, one’s SVO plays an important role in their prosocial behavior.

Empathy is associated with SVO in that it promotes other-oriented concerns and inhibits those that are more hedonistic and egocentric [36,37]. In an empathetic response, teachers step into their students’ shoes and share similar feelings, often eliciting the feeling of having an obligation to be fair. Furthermore, individuals’ social value preference affects their belief in a moral responsibility to ensure individual fairness. For instance, studies have shown that SVO significantly affects individuals’ tolerance for unfairness. Specifically, individuals with prosocial preferences are more intolerant of injustice and show greater levels of moral outrage in response. Prosocial preferences may further facilitate empathy’s positive benefits in promoting a sense of moral obligation. Thus, we posit the following hypothesis.

**Hypothesis 4.** Social value orientation moderates the effect of empathy on moral obligation.

As an important cognitive factor, moral obligation may interact with SVO and impact teachers’ fairness. Individuals with high moral standards who have internalized moral imperatives are more likely to be empathetic towards others, and thus are also more likely to enforce social norms they perceive as moral (e.g., fairness) [38]. For instance, individuals with higher moral standards are more willing to sacrifice their personal interests to punish unfair resource allocators and inclined to make fair resource allocations to others. Individuals with prosocial preferences are more concerned with equity [39], have a stronger sense of social responsibility, and show more altruistic behavior [40]. Consequently, for prosocial teachers, moral obligation is better translated as adherence to fairness rules. Thus, we propose the following hypothesis.

**Hypothesis 5.** Social value orientation moderates the effect of moral obligation on fairness behavior.

**Fig 1:**
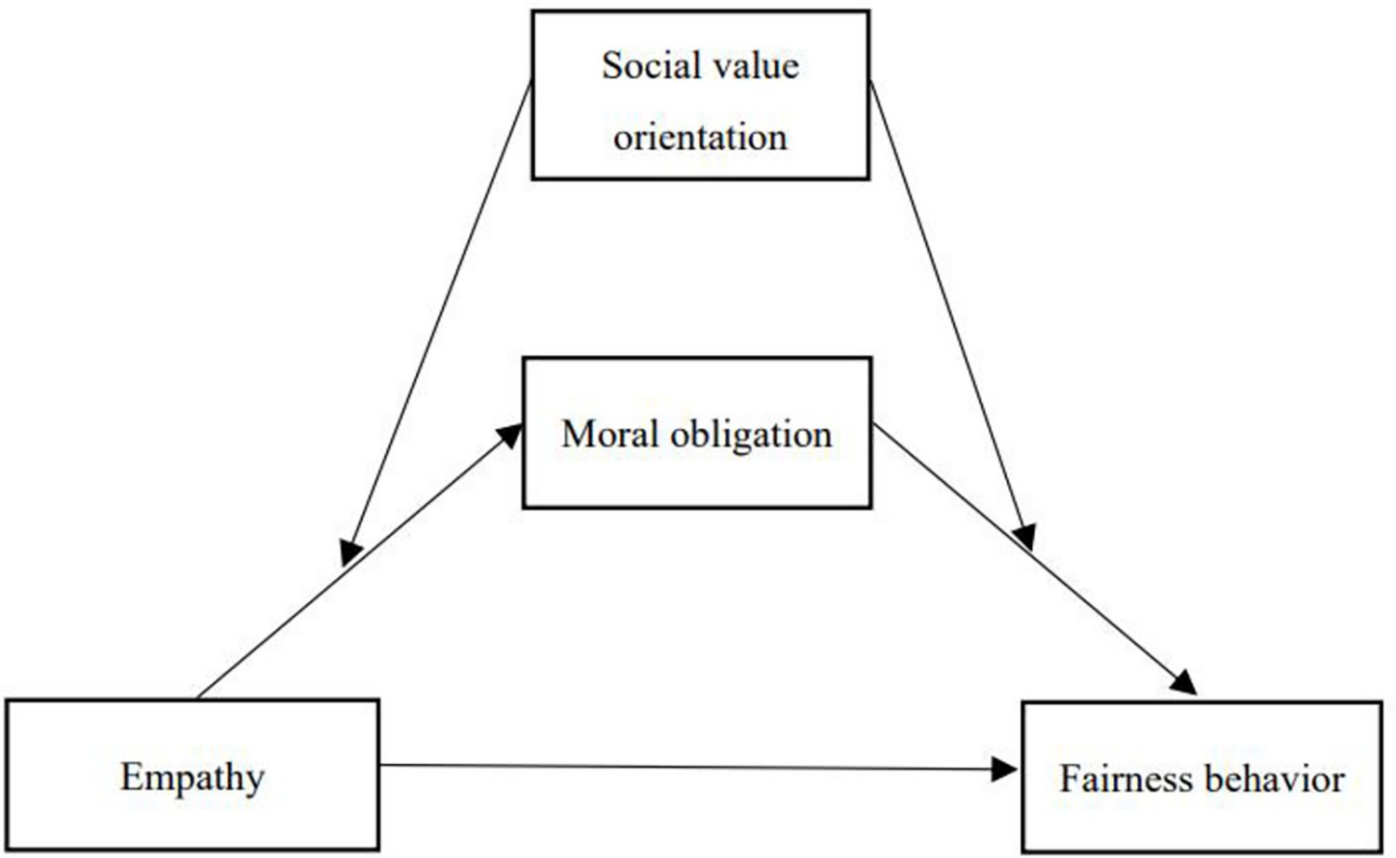
Proposed moderated mediation model.

In service of these hypotheses, we established a model to explore the relationship between empathy and teachers’ fairness behavior (see Fig 1).

## Method

### Participants and procedure

A total of 1,048 questionnaires were distributed to 11 primary and middle schools (six urban and five rural) in southern China, and 970 completed questionnaires were collected (response rate = 94.1%). Data from 74 teachers were removed, leaving 912 questionnaires that were usable for analysis (494 from females and 418 from males). The average age was 31.61 years (SD = 9.63) and 698 (76.5%) respondents had a bachelor’s degree. There were 479 teachers from urban schools (52.5%) and 433 from rural schools (47.5%). The measures were completed at their respective schools, when the participants were not in class. A research assistant organized respondents into small groups to fill out the questionnaire. Participants were free to ask the research assistant questions, if necessary. Each participant was asked if they would take part in a study on empathy and fairness. All provided written informed consent before filling out their questionnaire. The instrument included an explanation of the purpose of the survey and emphasized the voluntariness and anonymity of participation. The average time to complete the questionnaire was 20 minutes. All procedures were approved by the ethics committee at the authors’ institution.

### Measures

#### Empathy

Empathy was measured using the Measure of Empathy and Sympathy Scale developed by Vossen and revised for Chinese teachers by Wang [41]. The questionnaire has 12 items and measures three dimensions: cognitive empathy, affective empathy, and sympathy. The instrument uses a 5-point Likert-type scale ranging from 1 (“never”) to 5 (“always”). An example item is as follows: “I can often understand how people are feeling, even before they tell me.” Higher scores indicate greater levels of empathy. In the current study, the Cronbach’s alpha coefficient for the scale was 0.80.

#### Moral obligation

Moral obligation was measured using the moral obligation subscale of the Deontic Justice Scale developed by Beugre and revised for use in China by Zhang [42]. The moral obligation subscale is comprised of six items and uses a 5-point Likert scale ranging from 1 (“disagree”) to 7 (“strongly agree”). An example item is as follows: “I have a moral obligation to uphold the principles of fairness.” Higher scores indicate a greater sense of moral obligation. In the current study, the Cronbach’s alpha coefficient for the scale was 0.85.

#### Social value orientation

SVO was measured by the Triple Dominance Measure [43]. The scale consists of nine items; each has three options representing the distribution of different points. For example, Option A: 480 points for self and 80 points for other, Option B: 540 points for self and 280 points for other, and Option C: 480 points for self and 480 points for other. Option A represents the competitive choice, Option B the individualistic choice, and Option C the prosocial choice. Participants are classified as prosocial, individualistic, or competitive when at least six choices (out of nine) are consistent with one of the three orientations. Because both individualists and competitors have an egocentric focus in their outcome, in this research, they were combined to form the pro-self group [44]. This measurement has been proven to have good internal consistency, test-retest reliability, and ecological validity [45].

#### Teachers’ fairness behavior

Fairness behavior was measured by the Teacher Fairness Behavior Scale [46]. It consists of 12 items, with responses recorded on a 5-point Likert scale ranging from 1 (“totally disagree”) to 5 (“totally agree”). An example item is as follows: “I am equally patient with all my students.” Higher scores indicate more fairness behavior. In the current study, the Cronbach’s alpha coefficient for the scale was 0.90.

### Data analysis

Data analyses were conducted via SPSS 21. Harman’s single-factor test was used to assess the possibility of common method bias; no common method variance was detected (23.13% interpretation rate for the first factor < 40%) [47]. We used Models 4 and 58 of the PROCESS macro for SPSS to test the mediation and moderated mediation models, with 5,000 random sample bootstrapping confidence intervals [48]. All variables were standardized prior to being analyzed.

## Results

### Descriptive statistics of the variables

The means, standard deviations, and Pearson’s correlations are presented in Table 1. Empathy was positively correlated with moral obligation and fairness behavior. Moral obligation was positively correlated with fairness behavior.

**Table 1:** Descriptive statistics.

	*M*	*SD*	1	2	3	4
1.Gender	1.54	0.50	-			
2. Empathy	3.55	0.52	-0.01	1		
3. Moral Obligation	4.17	0.58	0.01	0.41***	1	
4.Teachers’ fairness behavior	4.16	0.55	0.06	0.28***	0.49***	1

Note: M = mean; SD = standard deviation.

*** p < 0.001.

### Analysis of moral obligation as a mediator

Model 4 of the PROCES macro was used to test the mediating effect of moral obligation (see Fig 1). Gender, age, and teaching experience were used as covariates. The results showed that empathy was positively associated with moral obligation (*b* = 0.41, *p* < .001), which in turn affected fairness behavior (*b* = 0.46, *p* < .001). However, the residual direct effect remained significant (*b* = 0.09, *p* < .01), showing that moral obligation partially mediated the correlation between empathy and fairness behavior (indirect effect = 0.19, 95% CI = 0.14 to 0.26). The final model accounted for 68% of the variance in fairness behavior. This finding supports Hypotheses 1 through 3.

### Analysis of social value orientation as a moderator

Model 58 of the PROCES macro was used to test the hypothesis regarding the moderation effect of SVO. SVO was dummy coded: prosocial tendency = 1 and pro-self tendency = 0. The “empathy × SOV” and “moral obligation × SOV” interaction terms being significant would have been evidence of SVO’s moderation effect (Frazier, Tix, & Barron, 2004). The results showed that empathy predicted moral obligation (*b* = 0.55, 95% CI = [0.46, 0.65], *t* = 11.16, *p* < 0.01) when SVO was coded as a dummy variable. The estimated coefficient indicated that SVO was also a predictor of moral obligation (*b* = 0.30, 95% CI = [0.18, 0.43], *t* = 4.72, *p* < 0.01), and the interaction term for empathy and SVO was significant (*b* = -0.23, 95% CI = [-0.36, -0.11], *t* = -3.70, *p* < 0.01). What’s more, moral obligation predicted fairness behavior (*b* = 0.55, 95% CI = [0.46, 0.65], *t* = 11.16, *p* < 0.01); SVO also predicted fairness behavior (*b* = 0.15, 95% CI = [0.03, 0.27], *t* = 2.38, *p* < 0.01), and the interaction term for moral obligation and SVO was significant (*b* = -0.18, 95% CI = [-0.30, -0.06], *t* = -2.96, *p* < 0.01). Thus, the results supported Hypotheses 4 and 5 (see Table 2).

**Table 2:** Coefficients for the tested moderated mediation model (*N* = 912).

	*R* ^2^	*F*	Coeff.	SE	95%CI
Moral obligation	0.21	38.14			
Constant			-0.38	0.13	-0.65 to -0.12
Gender			-0.03	0.06	-0.15 to 0.10
Age			0.01	0.01	-0.01 to 0.01
Teaching experience			0.06	0.04	-0.23 to 0.15
Empathy			0.55***	0.05	0.46 to 0.65
Social value orientation (SVO)			0.30***	0.06	0.18 to 0.43
Empathy×SVO			-0.23***	0.03	-0.35 to -0.11
Fairness behavior	0.27	46.55			
Constant			-0.43	0.13	-0.67 to -0.18
Gender			0.09	0.06	-0.02 to 0.21
Age			0.01	0.01	-0.01 to 0.01
Teaching experience			0.02	0.04	-0.06 to 0.10
Empathy			0.08*	0.03	0.02 to 0.14
Moral obligation			0.55***	0.05	0.45 to 0.65
Social value orientation (SVO)			0.15*	0.06	0.03 to 0.27
Moral obligation × SVO			-0.18**	0.06	-0.30 to -0.06

Note.SE = standard error; 95%CI = confidence interval with lower and upper limits. SVO was dummy coded as 1 = prosocial preference, 0 = proself preference

*p <0.05

**p <0.01

***p <0.001.

**Fig 2:**
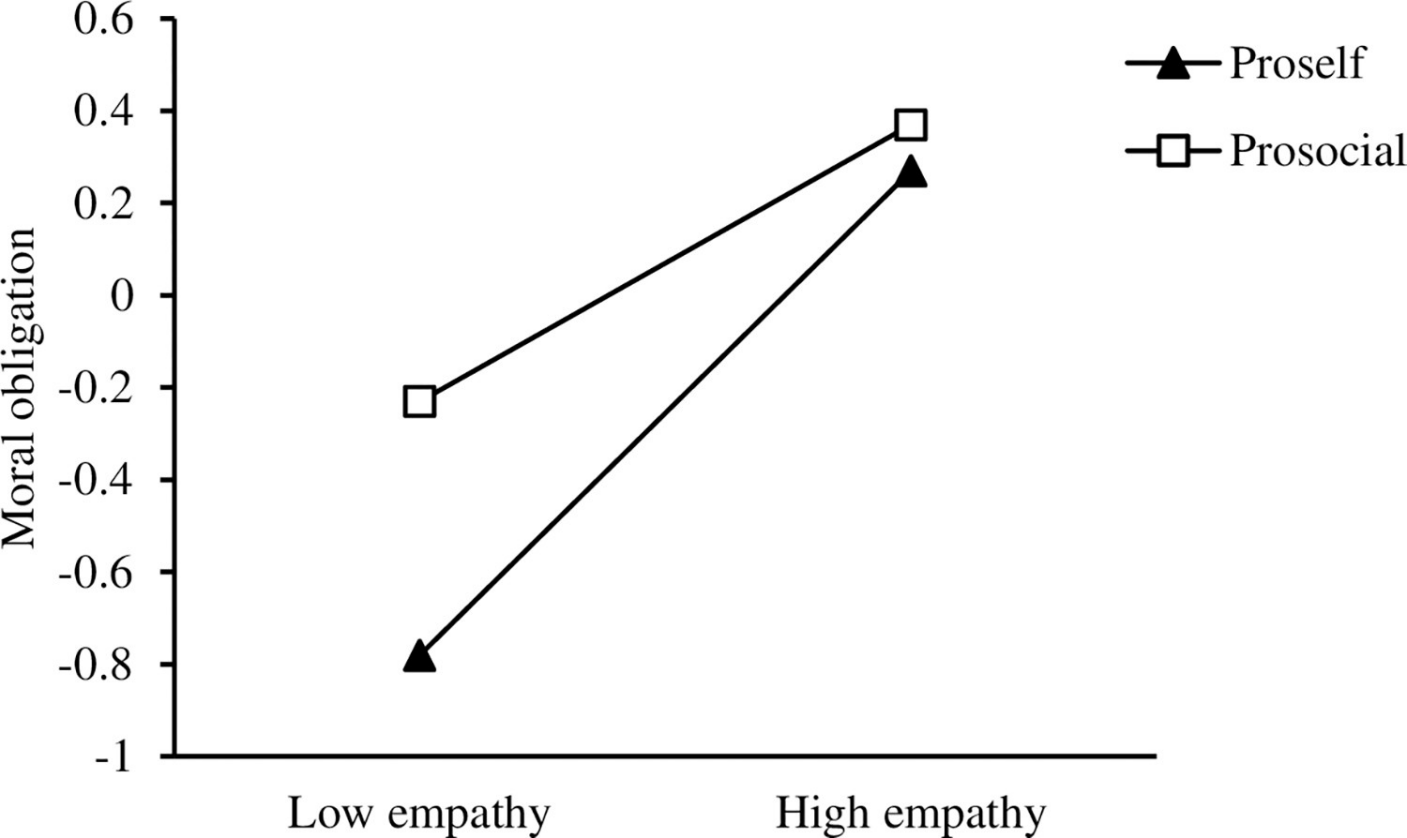
Interaction between empathy and social value orientation on moral obligation.

**Fig 3:**
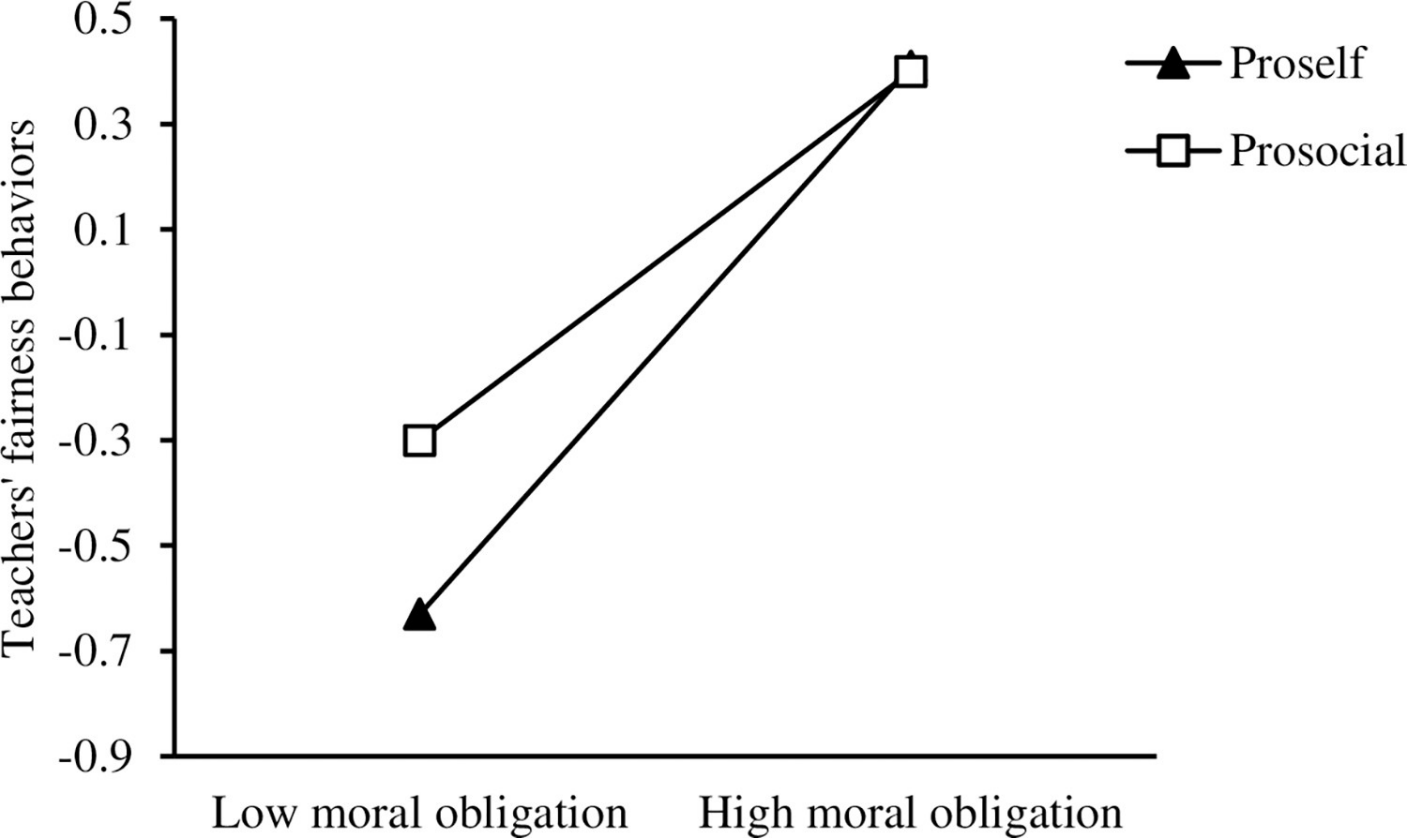
Interaction between moral obligation and social value orientation on teachers’ fairness behavior.

A simple slope analysis (see Fig 2) was conducted to indicate the separate relationships between empathy and moral obligation when SVO = 1 (i.e., a prosocial preference) and SVO = 0 (i.e., a pro-self preference; Aiken & West, 1991). For both groups, the moderating effect of SVO on the relationship between empathy and moral obligation was significant (for prosocial preference, *b* = 0.55, 95% CI = [0.46, 0.65], *t* = 11.26, *p* < 0.01; for pro-self preference, *b* = 0.32, 95% CI = [0.24, 0.39], *t* = 8.44, *p* < 0.01). The effect of empathy on moral obligation was weaker for teachers with a pro-self preference. The simple slope test (see Fig 3) also showed that moral obligation had a significant positive effect on teachers’ fairness behavior for those with both prosocial and pro-self tendencies (for prosocial preference, *b* = 0.55, 95% CI = [0.45, 0.65], *t* = 11.18, *p* < 0.01; for pro-self preference, *b* = 0.37, 95% CI = [0.29, 0.45], *t* = 9.46, *p* < 0.01). However, for teachers with pro-self tendencies, the relationship between empathy and moral obligation was significant but weaker. Moral obligation had more of a positive effect on fairness behavior in early childhood and primary school teachers with prosocial preferences.

## Discussion

This study contributes to the literature on empathy and fairness behavior by applying two theoretical perspectives to justice psychology: the empathy-altruism model and deontic theory of justice [49,50]. The results support the motivating effects of empathy and moral obligation on teachers’ fairness acts and a moderated mediation model in which SVO acts as an interpersonal trait, influencing the effects of empathy on moral obligation and moral obligation on fairness behavior. These results agree with the notion of a fairness act process in which empathy increases fairness behavior through motivation inspired by a sense of moral obligation. Our results also show that compared to teachers with pro-self tendencies, in teachers with prosocial tendencies, empathy has more of an effect on moral obligation and moral obligation more of an effect on fairness behavior. These results extend the research scope of organizational psychology to the field of education.

### Mediating effect of moral obligation

The results show that moral obligation played a mediating role between teachers’ empathy and their fairness behavior. First, according to the empathy-altruism model, in the field of education, teachers’ empathy can motivate them to behave fairly to improve students’ circumstances. In other words, empathy increases teachers’ circle of regard, and as a result, more students receive fair treatment. Second, according to deontic justice theory, individuals often feel a principled moral obligation to uphold norms related to justice. This finding indicates that emotion (i.e., empathy) and cognitive factors are important antecedents of fairness acts. Teachers who are more empathetic can better understand the negative effects of unfair treatment on students, thereby enhancing their perception of the importance of fairness [12]. This then increases their sense of responsibility and obligation to treat students fairly, leading to a greater likelihood of their doing so [51,52]. The current study confirmed the motivational role of deontic justice (e.g., moral obligation) in teachers’ fairness behavior from the perspective of justice actors and not just justice receivers.

### The moderating effect of SVO

The effect of empathy on a sense of moral obligation was found to be moderated by the interpersonal trait of SVO. In contrast to teachers with pro-self tendencies, the positive effect of empathy on moral fairness is stronger among teachers who are more prosocial. This result is consistent with prosocial people’s likelihood to make fair decisions [37]. For teachers with prosocial tendencies, their empathy enhances their belief in a responsibility to be fair and pay attention to the interests of all students [38].

SVO also plays a moderating role in the relationship between moral obligation and teachers’ fairness behavior. For teachers with prosocial preferences, motivation from a sense of moral obligation has more of an effect on fairness behavior. Previous studies have also confirmed that individuals with higher moral standards demonstrate more ethical and fair behavior [26].

The current study expands the understanding of the mechanism underlying the effect of empathy on teachers’ fairness behavior. From a theoretical standpoint, the results of this study will provide a new direction for building models of fairness acts in the field of education. In terms of clinical practice, the results will provide knowledge that will be useful for improving teachers’ ability to behave fairly. For instance, teachers should take part in workshops, training programs, and other forms of reinforcement that seek to motivate teachers to be fair. For those with a tendency to behave unfairly, it is important to enhance their empathy and cultivate a sense of moral obligation and prosocial preferences.

Despite the theoretical and practical implications discussed above, this study has the following limitations. Firstly, the cross-sectional design used in this research limits the identification of causal relationships among variables. Secondly, research data were collected through participants’ self-reporting. Since they knew that this was a study on empathy and fairness, they might have been more subjective in their responses, leading to self-report bias. Future research would benefit from collecting data from various respondents (e.g., students) and utilizing experimental methods to test the given model, such as by comparing empathetic and non-empathic initiation groups or designing a longitudinal method to test the relationships among the variables. Third, future research should explore the influence of other factors on teachers’ fairness behavior, such as environmental aspects (e.g., school climate). Fourth, as the current study was conducted with a sample of teachers from 11 primary and middle schools in Southern China, whether the findings discussed above can be generalized to teachers from other areas and types of schools remains undetermined.

## Supporting information

S1 File (ZIP)

S2 File (DOCX)
