# Supplementary material for: Empathy and teachers’ fairness behavior: The mediating role of moral obligation and moderating role of social value orientation
Source: PLoS One. 2022 Jun 9;17(6):e0268681. doi: 10.1371/journal.pone.0268681 (PMC9182229; doi:10.1371/journal.pone.0268681)
Supplement: S2 File — (DOCX) [file pone.0268681.s002.docx]

**Empathy**

| 1、即使别人不告诉我她（他）的内心情感，我往往也能够理解。 | 1 | 2 | 3 | 4 | 5 |
| --- | --- | --- | --- | --- | --- |
| 2、当朋友生气的时候，就算她（他）试图掩饰，我也能觉察。 | 1 | 2 | 3 | 4 | 5 |
| 3、当别人强颜欢笑时，我能看出她（他）实际上并不开心。 | 1 | 2 | 3 | 4 | 5 |
| 4、我善于识别她（他）人的真实情感。 | 1 | 2 | 3 | 4 | 5 |
| 5、当朋友表现出恐惧时，我也会有害怕的感觉。 | 1 | 2 | 3 | 4 | 5 |
| 6、当朋友伤心难过时，我也会变得伤心。 | 1 | 2 | 3 | 4 | 5 |
| 7、当朋友生气时，我也会觉得愤怒。 | 1 | 2 | 3 | 4 | 5 |
| 8、当朋友紧张时，我也会感到紧张。 | 1 | 2 | 3 | 4 | 5 |
| 9、当别人遭受不公平对待时我会有些同情他（她）。 | 1 | 2 | 3 | 4 | 5 |
| 10、当别人生病时，我会为他（她）担忧。 | 1 | 2 | 3 | 4 | 5 |
| 11、看到动物受到伤害时我会感到难过。 | 1 | 2 | 3 | 4 | 5 |
| 12、当朋友伤心时我会很担心他（她）。 | 1 | 2 | 3 | 4 | 5 |

Translation

1. Even if others don't tell me her (his) inner feelings, I can often understand.

2. When a friend is angry, even if she (he) tries to cover up, I can detect it.

3. When others force a smile, I can see that she (he) is actually not happy.

4. I am good at identifying her (his) true feelings.

5. When friends show fear, I also feel afraid.

6. When a friend is sad, I will become sad.

7. When a friend is angry, I feel angry too.

8. I feel nervous when my friends are nervous.

9. I sympathize with others when they are treated unfairly

10. When someone is ill, I will worry about him or her.

11. I feel sad when I see animals being hurt.

12. When a friend is sad, I will worry about him (her)

**Moral obligation**

| 1、我有公平对待他人的道德义务。 | 1 | 2 | 3 | 4 | 5 |
| --- | --- | --- | --- | --- | --- |
| 2、公平待人应该是每个人的道德义务。 | 1 | 2 | 3 | 4 | 5 |
| 3、尊重和有尊严地对待人是一种道德义务。 | 1 | 2 | 3 | 4 | 5 |
| 4、我在道义上有义务维护公平原则。 | 1 | 2 | 3 | 4 | 5 |
| 5、对我来说，确保其他人得到公平对待是很重要的。 | 1 | 2 | 3 | 4 | 5 |
| 6、对我来说，公平待人是一种道德义务。 |  |  |  |  |  |

1. I have a moral obligation to treat others fairly.

2. Treating people fairly should be everyone's moral obligation.

3. Treating people with respect and dignity is a moral obligation.

4. I have a moral obligation to uphold the principle of fairness.

5. It's important for me to be treated fairly.

6. For me, treating people fairly is a moral obligation.

**Social value orientation**

如果你和一个陌生人正在分配一些点数（点数是对你和对方都很重要的东西，如金钱），下列ABC代表不同的分配方案。你和他都将在这些方案中选择，对方无法知道你的选择。你得到的点数数值越大，对你自己越有利;而对方的点数数值越大，对对方越有利。你的选择会影响到你自己和对方最终所获得的收益。请你根据自己内心的真实想法对下列9个情境做出选择。

**1、情境一 [三选一]**

| A方案(你将得到480，对方将得到80)  B方案(你将得到540，对方将得到280) C方案(你将得到480，对方将得到480)  **2、情境二 [三选一]**   \| A方案(你将得到560，对方将得到300)  B方案(你将得到500，对方将得到500) C方案(你将得到500，对方将得到100) \| \| --- \| |
| --- | --- |

**3、情境三 [三选一]**

| A方案(你将得到520，对方将得到520)  B方案(你将得到520，对方将得到120) C方案(你将得到580，对方将得到320)  **4、情境四 [三选一]**   \| A方案(你将得到500，对方将得到100)  B方案(你将得到560，对方将得到300) C方案(你将得到490，对方将得到490) \| \| --- \| |
| --- | --- |

**5、情境五 [三选一]**

| A方案(你将得到560，对方将得到300)  B方案(你将得到500，对方将得到500) C方案(你将得到490，对方将得到90)  **6、情境六 [三选一]**  A方案(你将得到500，对方将得到500)  B方案(你将得到500，对方将得到100) C方案(你将得到570，对方将得到300) |
| --- |

**7、情境七 [三选一]**

| A方案(你将得到510，对方将得到510)  B方案(你将得到560，对方将得到300) C方案(你将得到510，对方将得到110) |
| --- |

**8、情境八 [三选一]**

| A方案(你将得到550，对方将得到300)  B方案(你将得到500，对方将得到100) C方案(你将得到500，对方将得到500) |
| --- |

**9、情境九 [三选一]**

| A方案(你将得到480，对方将得到100)  B方案(你将得到490，对方将得到490) C方案(你将得到540，对方将得到300) |
| --- |

1.

A.Option A: 480 points for self and 80 points for the other person

B.Option B: 540 points for self and 280 points for the other person

C.Option C: 480 points for self and 480 points for the other person

**2.**

A.Option A: 560 points for self and 300 points for the other person

B.Option B: 500 points for self 500 points for the other person

C.Option C: 500 points for self and 100 points for the other person

**3.**

A.Option A: 520 points for self and 520 points for the other person

B.Option B: 520 points for self and 120 points for the other person

C.Option C: 580 points for self and 320 points for the other person

**4.**

A.Option A: 500 points for self and 100 points for the other person

B.Option B: 560 points for self and 300 points for the other person

C.Option C: 490 points for self and 490 points for the other person

**5.**

A.Option A: 560 points for self and 300 points for the other person

B.Option B: 500 points for self and 500 points for the other person

C.Option C: 490 points for self and 90 points for the other person

**6.**

A.Option A: 500 points for self and 500 points for the other person

B.Option B: 500 points for self and 100 points for the other person

C.Option C: 490 points for self and 90 points for the other person

**7.**

A.Option A: 510 points for self and 510 points for the other person

B.Option B: 560 points for self and 300 points for the other person

C.Option C: 510 points for self and 110 points for the other person

**8.**

A.Option A: 550 points for self and 300 points for the other person

B.Option B: 500 points for self and 100 points for the other person

C.Option C: 500 points for self and 500 points for the other person

**9.**

A.Option A: 480 points for self and 100 points for the other person

B.Option B: 490 points for self and 490 points for the other person

C.Option C: 540 points for self and 300 points for the other person

**Teachers’ fairness behavior**

| 1、课堂上，我会让每位学生都有机会表达自己的观点。 | 1 | 2 | 3 | 4 | 5 |
| --- | --- | --- | --- | --- | --- |
| 2、优秀的学生与表现不好的学生一起做错事时，我对他们的惩罚是一样的。 | 1 | 2 | 3 | 4 | 5 |
| 3、总体上，我会顾及班里每个学生的感受。 | 1 | 2 | 3 | 4 | 5 |
| 4、无论什么事情，我对优秀的学生不会有任何偏袒。 | 1 | 2 | 3 | 4 | 5 |
| 5、在批改作业时，我对不同学生的评分标准是一样的。 | 1 | 2 | 3 | 4 | 5 |
| 6、我努力保证每位学生都能参与到学习过程中来。 | 1 | 2 | 3 | 4 | 5 |
| 7、不管和哪位学生讨论问题，我对他们都一样耐心。 | 1 | 2 | 3 | 4 | 5 |
| 8、我不会对学生干部格外照顾。 | 1 | 2 | 3 | 4 | 5 |
| 9、我总是让自己和每位学生都有机会互动。 | 1 | 2 | 3 | 4 | 5 |
| 10、我总是严格按照评分标准来批改学生试卷。 | 1 | 2 | 3 | 4 | 5 |
| 11、我能关心班级里的每个学生。 | 1 | 2 | 3 | 4 | 5 |
| 12、在课堂上，当少数学生不理解的时候，我会停下来重复。 | 1 | 2 | 3 | 4 | 5 |

1. In class, I will give every student the opportunity to express their views.

2. When a good student does something wrong with a bad student, I punish them the same.

3. On the whole, I will take into account the feelings of every student in the class.

4. No matter what happens, I won't be partial to excellent students.

5. When correcting homework, I grade different students according to the same standard.

6. I try to ensure that every student can participate in the learning process.

7. No matter which student I discuss problems with, I am as patient with them.

8. I will not take extra care of student cadres.

9. I always give myself the opportunity to interact with every student.

10. I always mark students' papers in strict accordance with the scoring standards.

11. I can care about every student in the class.

12. In class, when a few students don't understand, I will stop and repeat,
